# Rapid adaptation of first contact physiotherapy services for musculoskeletal patients in the UK and Australia during the COVID-19 pandemic: A multiple case study

**DOI:** 10.1177/13558196261452621

**Published:** 2026-05-21

**Authors:** Oluwatoyin Adenike Adeniji, Theopisti Chrysanthaki, Evangelos Pappas, Magdalena Zasada, Karen Stenner, Nicola Carey

**Affiliations:** 1 School of Health Sciences, Faculty of Health and Medical Sciences, 3660 University of Surrey, Guildford, UK; 2 School of Medical, Indigenous and Health Sciences, Faculty of Science Medicine and Health, University of Wollongong, Wollongong, NSW, Australia; 3 School of Health and Biomedical Sciences, 5376 Royal Melbourne Institute of Technology University, Melbourne, VIC, Australia; 4 Sydney School of Health Sciences, The University of Sydney, Sydney, NSW, Australia; 5 Centre for Rural Health Sciences, 7709 University of the Highlands and Islands, Scotland, UK

**Keywords:** rapid adaptation, first contact physiotherapy, continuity of care

## Abstract

**Background:**

The disruption of health services by the COVID-19 pandemic prompted the WHO to advocate for rapid adaptation of healthcare. Little is known about the specific rapid adaptation strategies deployed in first contact physiotherapy services (FCPS) and how they were experienced by providers and patients.

**Study Aim:**

To explore views and experiences of physiotherapists and key stakeholders on the rapid adaptation of FCPS during the COVID-19 pandemic in the UK and Australia.

**Methods:**

A multiple case study design was employed across four case sites; UK (n = 2), Australia (n = 2), involving online semi-structured interviews with 22 participants in 2024 and document review. A case-based approach using framework analysis was applied, drawing on the public health emergency framework of readiness, responsiveness, and sustainability, alongside the consolidated framework for implementation research (CFIR).

**Results:**

Gaps in readiness for adaptation, particularly in terms of planning and resources, were evident across FCPS in both countries. However, the findings highlight FCPS as an adaptable service, with strategies implemented within the first three to six months following the declaration of COVID-19 as a pandemic. The most notable adaptation variation was in telehealth, where alignment with service contexts influenced implementation and ongoing sustainability. New challenges not fully captured within CFIR constructs emerged, including the initial lack of acceptability, particularly related to telehealth, and the unpredictable nature of the pandemic.

**Conclusion:**

To strengthen FCPS preparedness for the future, policies should proactively equip services with the resources and capabilities required to manage emergencies and associated contingencies, while accounting for contextual variations. Such forward planning would support an effective response and the long-term integration of beneficial adaptations, thereby strengthening service resilience.

## Introduction

The COVID-19 pandemic posed unprecedented challenges to health systems globally, including widespread disruptions to health services, underscoring the need for rapid adaptation to maintain essential care.^1^ Rapid adaptation refers to the quick implementation of interventions, including structural and technological changes, to address urgent healthcare needs.^2–4^ This approach contrasts with traditional evidence-based implementation, which can take up to 17 years.^4–6^ Rapid adaptation ensures healthcare systems can quickly adjust to evolving challenges while maintaining service delivery.^7^ Within this context, First Contact Physiotherapy Services (FCPS) demonstrated adaptability to the rapidly evolving circumstances brought on by the COVID-19 pandemic.^8–10^ FCPS act as the initial point of contact for patients with musculoskeletal (MSK) conditions^11^ which affect approximately 1.71 billion people globally.^12^ FCPS are implemented at various access points within public health systems in various countries including the UK and Australia.^11^ In the UK, FCPS are well-integrated into primary care settings, allowing patients direct access to physiotherapy services through their general practice (GP).^13,14^ This model fosters a streamlined approach to MSK care, facilitating faster responses and reducing general practitioner workload.^15^ In contrast, Australia’s FCPS, sometimes referred to as primary contact physiotherapy, vary by region and are typically integrated into emergency departments (EDs) as a response to the high volume (30%) of MSK presentations in this setting.^16^ Despite the differing implementation contexts, both models ultimately share the same purpose: responding to system pressures and capacity challenges, particularly those related to MSK presentations and demand, while converging on common aims such as improving timely access, efficiency, and safety across MSK pathways, strengthening multidisciplinary integration and promoting advance practice scope.^16–18^

While a recent study reported changes in FCPS in the UK and Australia during the COVID-19 pandemic,^9^ research is lacking on the contextual complexities influencing rapid adaptation and preparedness in these settings. Differences in how FCPS are implemented across various settings may have affected the adaptability of these systems during the pandemic. Additionally, there is limited understanding of what constitutes responsive strategies for FCPS. A comprehensive examination of FCPS’ rapid adaptation, including contextual variations, is therefore essential in developing resilient FCPS capable of maintaining quality care under pressure. This study draws on the public health emergency framework, which emphasises readiness, responsiveness, and sustainability as key components that underpin effective emergency response.^19^ In this study, these components are conceptualised as service-level outcomes. Readiness refers to the healthcare system’s ability to implement necessary adaptations promptly in response to urgent situations, considering system capacity, available resources, and organisational willingness, thereby reflecting the overall preparedness of services.^20^ Responsiveness assesses the system’s ability to meet patient expectations and needs through adapted strategies.^21^ Sustainability, in turn, evaluates the capacity of the system to maintain beneficial adaptations beyond the immediate crisis, providing insight into long-term improvements and the enduring effectiveness of emergency response measures.^22^

Although implementation frameworks are instrumental in guiding the adoption and outcomes of evidence-based interventions, their tools are not designed specifically for public health emergencies, where emerging evidence becomes important. Nevertheless, they remain valuable for identifying the features of implementation. The Consolidated Framework for Implementation Research (CFIR)^23^ is one such widely used tool for explaining factors that influence implementation outcomes. In this study, CFIR was used to explore factors influencing readiness, responsiveness, and sustainability of FCPS’ rapid adaptation. CFIR comprises five domains: innovation, individual characteristics, inner setting, outer setting, and implementation process, encompassing 39 constructs influencing implementation. The innovation domain outlines the features of strategies or interventions implemented. Individual characteristics pertain to personal traits and roles while the inner setting refers to the environment where strategies or interventions are implemented. The outer setting indicates external factors that influence the inner setting while the process denotes the actions involved in carrying out an intervention or strategy.^23^ Given the importance of rapid adaptation in maintaining FCPS during crises, and the similarity in overarching implementation aims across varied contexts, examining how structurally distinct models adapted under pressure enables insights into how contextual factors shape readiness, responsiveness, and sustainability. This study aims to identify both context-specific and shared factors across FCPS that could contribute to more resilient and responsive service delivery by exploring the views and experiences of physiotherapists and key stakeholders on the rapid adaptation of FCPS during the COVID-19 pandemic in the UK and Australia.

## Methods

### Study design

A multiple case study design using four case sites, with data collected through semi structured interviews and organisational documents, was employed to explore rapid adaptation of FCPS during the COVID-19 pandemic. Data collection included interviews with FCPS physiotherapists and other stakeholders, and a review of relevant documents from each UK and Australian case site. Ethics approval for the UK component was granted by the Health Research Authority (HRA) (23/NW/0200). For the Australian component, ethics approval was obtained from the University of Wollongong Human Research Ethics Committee (2023/ETH00538), with additional sites-specific assessment for governance approval granted (2023/STE00836). This paper follows the Standards for Reporting Qualitative Research.^24^

### Researchers’ characteristics and reflexivity

OA, a doctoral researcher and MSK physiotherapist with 15 years of experience, was responsible for recruiting participants and conducting the interviews. OA had no prior relationship with participants and had not practiced at the case sites. Data were collected online, and OA used a reflexive journal to guide interview improvements and reduce unconscious bias, including during analysis.^25^ In addition, the analysis was conducted with two members of the team who are experienced qualitative researchers (TC and KS).

### Sampling and recruitment

This study is part of a wider study examining the rapid adaptation of FCPS during the COVID-19 pandemic in the UK and Australia.^26^ Earlier study phases utilised a cross-sectional survey of physiotherapists in FCPS roles in the UK and Australia to identify changes implemented in FCPS, including the timeline,^9^ providing the focus and sampling frame for this study.

#### Case sites

Four case sites, two each in the UK and Australia, were purposively selected based on contrasting changes in health service delivery identified in the earlier study,^9^ particularly differences in the perceived use of telehealth versus in-person care (i.e., increased use versus similar levels of use during the pandemic). Regarding case sites sample size, empirical evidence suggests that saturation occurs at ten to twelve interviews when considering an in-depth interview, however, more is required for a heterogeneous sample with a higher degree of saturation,^27^ such as this study. Taking a pragmatic approach, the aim was to recruit 40 participants, with 10 participants from each case site.

#### Physiotherapists in FCPS role

Physiotherapists from the earlier study who had expressed interest in future research and provided their email addresses (*n* = 15) were contacted. Following organisational approval, four physiotherapists who served as access points to the case sites were selected. These physiotherapists consented to participate in the study, along with additional physiotherapists from their organisations who were identified through them (*n* = 6). Eligibility included physiotherapists practicing in first contact roles, primarily managing patients with MSK conditions within UK National Health Service GP settings or Australian government hospital EDs, during the pandemic.

#### Stakeholders

Stakeholders, those whose work influenced or was affected by healthcare decisions, were identified using a snowball sampling approach. Physiotherapists from the four case sites assisted in identifying other potential participants including doctors, nurses, managers, allied health professionals, administrative and support staff (*n* = 16). Eligibility included individuals with knowledge of FCPS and its operation during the COVID-19 pandemic. After participants had been nominated, OA formally recruited and obtained consent. Prospective participants received a participant information sheet and consent form via email and were given 2 weeks to decide on their participation.

### Data collection and instruments

One-to-one online semi-structured interviews were conducted between September 2023 and March 2024, lasting an average of 70 min (ranging from 50 to 90 min). Interviews were conducted by OA in English via MS Teams or Zoom using an interview guide (Online Supplemental file 1). The interview guide was piloted with a physiotherapist in a FCPS role prior to the interview. Based on feedback, the authors reviewed the length and content of the interview, refining the interview guide by removing duplicate questions, enhancing clarity of terms, and making the questions more concise and focused. To enhance contextual understanding of the participating FCPS, organisational documents were also reviewed. Physiotherapists and managers from all four case sites were asked to provide documents outlining decisions, processes, and outcomes of implemented changes during the COVID-19 pandemic, as well as service planning before and/or during the pandemic. Documents were requested immediately after the interviews and were emailed to OA.

### Case sites and participants

Four case sites were included in this study. Two from the UK (cases 1 and 2) and two from Australia (cases 3 and 4). Cases 1 and 2 represent GPs, where FCPS were integrated within primary care networks in the East Midland and South-East regions of England. Cases 3 and 4 represent teaching hospitals with ED where FCPS were based, located in New South Wales, Australia. An initial sample size of 40 was intended. Of the 37 individuals who expressed interest in the study, 28 provided consent, and 22 participated (Case 1: *n* = 3; Case 2: *n* = 7; Case 3: *n* = 7; Case 4: *n* = 5), reflecting organisational capacity and interest. Participants are referred to by a code in this paper. Data saturation was reached after eight interviews in each country, and subsequent transcripts yielded repetitive patterns. The organisational documents were used to validate contextual information about the case sites provided by participants during interviews (See Online Supplemental Material 2 and 3 for case-site/participants’ details and the sampling flow chart).

### Data analysis

Interview audio recordings were transcribed verbatim and anonymised with assigned codes. OA, TC and KS reviewed the transcripts for accuracy and to familiarise themselves with the data. The transcripts were then imported into NVivo version 14 for further analysis. OA developed a coding framework based on the concepts of readiness, responsiveness, and sustainability, incorporating the CFIR.^23^ The coding framework allowed flexibility, with non-aligned themes coded separately and retained as distinct findings when they offered meaningful insights, thereby maintaining both theoretical rigor and contextual sensitivity. This was shared with the author team for feedback and consensus. Minor adjustments were suggested to improve contextual variation. OA, and two members of the team (TC, and KC) then worked to finalise the coding content and reach an agreement.

A case-based analysis, embracing Stake’s cross-case analytical strategy, to iteratively summarise cases around a deductive theme was adopted.^28^ Framework analysis^29^ was used to draw out cross-cutting themes. Coding consistency was facilitated by OA, TC and KS independently coding one interview from each of the four case sites. This step also aimed to enhance the credibility and validity of the research findings. To evaluate intercoder reliability, Cohen’s kappa statistic was used, which accounts for the possibility of agreement by chance. The analysis yielded a Cohen’s kappa coefficient of 0.62, indicating moderate agreement.^30^ For instance, the coders initially disagreed on the categorisation of facilitators and responsiveness. To address these discrepancies, a meeting was held where OA, TC and KS reviewed the ambiguous categories, discussed differing interpretations, and resolved key differences. Following the inter-coding process, OA proceeded to analyse the remaining interview data, using NVivo version 14. Organisational documents received from all four case sites were reviewed only to support contextual understanding, as formal document analysis was not feasible. This was due to the diverse nature of the documents received, which primarily outlined service plans (i.e., service structure, staffing levels, and patient population). It was apparent that the service plans reviewed were not designed to capture the changing and evolving aspects of service delivery during the pandemic, which were central to the study aims.

## Results

**Figure 1.**
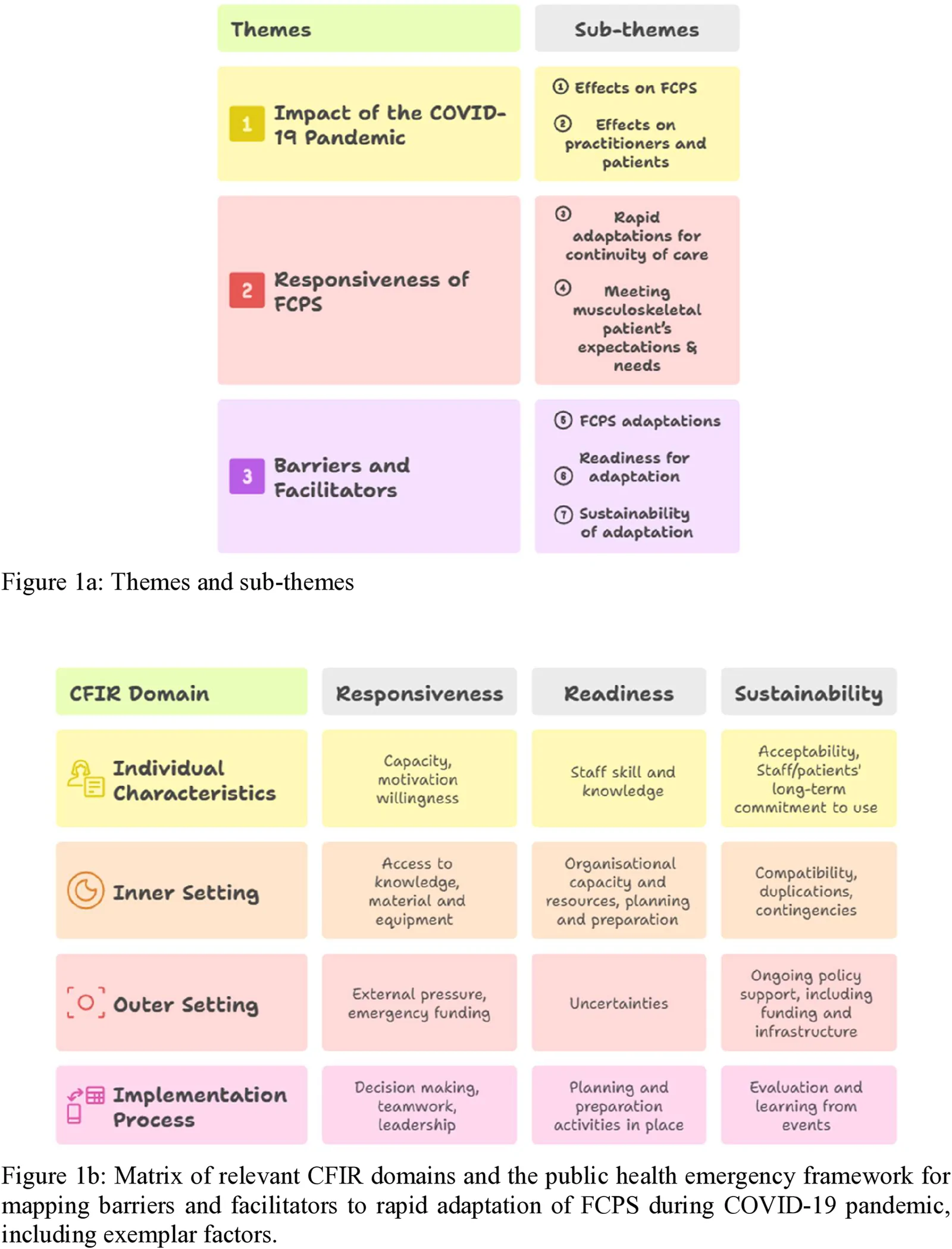
Themes and matrix.

The findings of this study are presented here as three main themes (Figure 1a): the impact of the COVID-19 Pandemic on FCPS, FCPS responsiveness to the COVID-19 Pandemic, and barriers and facilitators of FCPS rapid adaptation. The impact of pandemic went beyond the established framework, offering broader insights into the contextual and interrelated factors that may promote future service preparedness. The responsiveness of FCPS highlighted the service level outcomes and the capacity of the service to respond rapidly, through the implementation of adapted strategies. Barriers and facilitators to adaptation mapped onto the public health emergency and CFIR frameworks, as illustrated in the matrix presented in Figure 1b. Together, these themes offer insights into the impacts, processes, strategies and factors influencing rapid adaptation in FCPS during the COVID-19 pandemic. (see Online Supplemental file 4 for codes and additional quotes).

### Impact of the COVID-19 pandemic on first contact physiotherapy service

#### Effect on FCPS delivery

The findings revealed both similarities and differences in how the COVID-19 pandemic impacted FCPS across the four case sites. In the UK GP settings (cases 1 and 2), the pandemic caused significant service disruption, ranging from complete closure to minimal service provision during the first three to 6 months of the pandemic:

From March to September, six months basically, there wasn’t any FCPS going on in our GP, only urgent cases were addressed, like cauda equina, an infected knee, or significant osteoarthritis flare-ups. (Case 2 GP (Physiotherapist) R1).

In contrast, participants from the ED settings (cases 3 and 4) discussed how they managed to maintain continuous FCPS delivery throughout the pandemic, albeit with notable reductions in MSK patient presentations. Physiotherapists and operational managers attributed this decline in ED settings to lockdown measures and heightened public fear of contracting COVID-19, especially with a perceived influx of COVID-infected patients into ED:

The COVID situation created a lot of buzz, causing fear coupled with restrictions, this played a role in the decreased presentation of MSK patients to the ED” (Case 3 ED (Physiotherapist) R3).

#### Effects on practitioners and patients

Our analysis showed that physiotherapists from various settings perceived that the COVID-19 pandemic had heightened pre-existing challenges, such as burnout, and created some new ones, such as a sense of working beyond their scope of practice. In case 3, hospital ED, physiotherapists specialising in MSK care were reassigned to treat COVID-infected patients with respiratory conditions. Similarly, in Case 2 (a UK GP setting), a physiotherapist reported being redeployed to community services to support district nurses with patient care, an experience that was common across their primary care network. However, this redeployment was met with challenges, as many physiotherapists felt they were not skilled for the tasks, expressing being less satisfied with the assigned duties:

Physiotherapists were redeployed to the community to help, but they kind of struggled because they were not skilled in nursing care. (Case 2 GP (Practice nurse) R3).

Post-lockdown, physiotherapists faced increased workloads and complex patient presentations, compounded by staff shortages. In case 1 (GP), MSK patients experienced worsening symptoms due to limited healthcare access during the lockdown, particularly in economically deprived areas where public health services were already overstretched. A similar pattern was observed in the ED, where FCPS experienced both higher patient numbers and more complex cases after the lockdown period.

We saw an upswing of musculoskeletal complaints and complex cases in our ED because people couldn't get into their primary care provider. (Case 4 ED (Physiotherapist) R1).

Additionally, new occupational health hazards emerged. In ED settings (cases 3 and 4), physiotherapists highlighted exposure to COVID-19 infection as a major risk, leading to illness and staff shortages. In GP settings (cases 1 and 2), physiotherapists reported that ergonomic challenges arose from increased telehealth use, such as MSK pain due to prolonged desk work:

When you’re on a phone or on a video call, you’re just sitting a lot more, and that results in so much body ache. (Case 2 GP (Physiotherapist) R1).

### First contact physiotherapy services responsiveness to COVID-19 pandemic

#### Rapid adaptations for the continuity of care

The COVID-19 pandemic prompted rapid changes in FCPS, introducing new service models, providing insights into strategies that worked well (Table 1). In UK GP settings (cases 1 and 2), strategies such as telehealth adoption for appointment booking, e-consultation, and sharing self-management advice were implemented by September 2020. Other measures included the use of personal protective equipment (PPE) and a transition to a hybrid care model, in which two appointments (telehealth and in-person consultations) replaced the previous single in-person appointment system. Despite occurring 6 months into the pandemic, participants still considered these adaptations rapid:

So when the FCPS in our GP re-opened, everybody was just doing telephone consultation; it was quickly implemented. (Case 1 GP (Receptionist) R3).

**Table 1.** Key adaptations and strategies that worked well for FCPS during the COVID-19 pandemic.

Key changes	Case site 1 Primary care (GP) UK	Case site 2 Primary care (GP) UK	Case site 3 Emergency Department (Hospital) Australia	Case site 4 Emergency Department (Hospital) Australia
Initial changes March to September 2020				
Increased staffing and or redeployment	✓	✓	✓	✓
New services implemented	✓ (New FCPS within PCN)	✗	✓ (New weekend MSK service)	✓ (New rapid MSK assessment & discharge)
Online booking system	✓	✓	✗	✗
Virtual diary	✓	✓	✗	✗
Virtual consultation and self-management intervention through telehealth	✓	✓	✗	✗
Use of PPE	✓	✓	✓	✓
Service restructuring	✓	✓	✓	✓
Subsequent changes October 2020 and beyond				
Hybrid care	✓	✓	✗	✗
Removal of waiting area	✗	✓	✗	✗
Revert back to face to face/in person care	✓	✓	✗	✗
Commenced building of infrastructure-physical space	✗	✗	✗	✓
Revert back to prior service structure	✗	✓	✓	✓
Commenced staff training on extended scope of practice	✗	✗	✓	✗

Strategies that worked well	Primary care (GP) UK	Emergency Department (Hospitals) Australia
Local decision making (health services level and by management and practitioners)	✓	✓
Quick access to knowledge and information	✓	✓
Availability of PPE	✓	✓
Staff upskill and self-learning	✓	✓
The use of telehealth for various aspects of care delivery	✓	✗
Hybrid care	✓	✗
Rapid evaluation, learning and continuous improvement	✓	✓
Teamwork & interpersonal relationship	✓	✓
Personalised care (patient centred care)	✓	✓
Redeployment of staff into FCPS	✗	✓
Rapid and successive planning, preparation and adaptation	✓	✓
Emergency funding	✓	✓
Triage system	✓	✓
Availability of COVID vaccine	✓	✓

GP: General practice; FCPS: First contact physiotherapy services; PCN: Primary Care Network; MSK: Musculoskeletal; PPE: Personal Protective Equipment; ✓: Yes; ✗: No

In contrast, ED settings (cases 3 and 4) exhibited continuity with pre-pandemic practices, i.e., in-person care, however with PPE. Technology was used for limited purposes, such as triaging and occasionally sharing self-management advice. Early into the pandemic (April/May 2020), case 3 initiated upskilling and redeployment efforts, eventually introducing weekend FCPS services by September 2020 to enhance access. Case 4 took a unique approach with the implementation of a rapid assessment and discharge unit for ED MSK patients. This unit, staffed by a multidisciplinary team, worked extended hours to maintain continuity of care and improve patient flow, particularly for older patients with chronic MSK conditions. Participants across all case sites emphasised the need for preparedness and structured plans for future crises. For instance, an operational manager from case 3 highlighted the benefits of the availability of a competency framework which facilitated the upskilling and redeployment of physiotherapists to the ED, suggesting that having a guideline available for emergency situations would support adaptive readiness:

It would be helpful to have a guideline and policy early on to handle crises without pressure. (Case 4 ED (Physiotherapist) R2).

#### Meeting MSK patient’s expectations and needs

The COVID-19 pandemic posed significant challenges to meeting MSK patients’ expectations due to stay-at-home policies in both the UK and Australia. In cases 1 and 2, physiotherapists and nurses reported that patients initially had limited choices regarding their care options and were largely restricted to virtual consultations. However, as lockdown restrictions eased and GP services resumed, patients had greater freedom to choose between telehealth, in-person, or hybrid care models. In addition, MSK telehealth services were tailored to patients’ needs. This included providing online resources and exercise videos for remote care. Although decisions regarding management and the mode of MSK care were informed by practitioners’ judgement, patients eventually regained autonomy, including the choice to access care virtually or attend in person. While cases 3 and 4 remained open to MSK patients during the pandemic, physiotherapists in case 4 emphasised the need for prompt and fear-free accessibility for responsive care. A proposed solution was accelerating the integration of FCPS into Australian primary care. They noted that this model could enable patients to access physiotherapists directly through GPs for more responsive care:

The Australian Physiotherapy Association is already pushing for the integration of FCPS in GP, but implementing these services requires government funding. If we can make this happen, MSK patients would have the convenience of accessing physiotherapists directly through their GP just like we do here. And I think the fear of attending the GP was lesser during the COVID compared to the ED (Case 4 ED (Physiotherapist) R2).

### Barriers and facilitators of FCPS rapid adaptation

This theme highlights the factors that influenced the rapid adaptation of FCPS during the COVID-19 pandemic. Based on CFIR, factors relating to individual characteristics, inner setting, implementation process and outer setting served as both barriers and facilitators.^23^ These factors were categorised into three subthemes: adaptations in FCPS, readiness for adaptation, and sustainability of adaptation strategies (see Online Supplemental material 5).

#### Adaptations in first contact physiotherapy services delivery

Individual characteristics such as staff commitment and career growth emerged as both facilitators and barriers during the pandemic. In cases 3 and 4, physiotherapists and managers emphasised staff dedication, especially from physiotherapists redeployed from other hospital units to the ED. Their willingness to upskill rapidly and adapt ensured continued patient care. Some viewed redeployment as an opportunity for career progression, gaining experience in ED roles and transitioning into them permanently:

So, staff were upskilled and sent to the ED, so that's why I started working here. I was just happy to stay here since. (Case 3 ED (Physiotherapist) R4)

Similarly, in case sites 1 and 2, career development was evident, though experience varied as they saw a reverse redeployment, with physiotherapists moved from FCPS to community services. Participating physiotherapists’ accounts of others in FCPS roles within the same primary care network revealed that while some physiotherapists struggled with working outside their usual scope of practice and looked forward to returning to FCPS, others appreciated the slower pace of community services. This led to a few people permanently transitioning out of FCPS due to concerns over intense workloads and potential future redeployment:

One or two physiotherapists within our primary care network actually left FCPS, preferring the slower pace of community services. (Case 2 GP (Physiotherapists) R1).

In terms of the inner setting, all case sites highlighted the dual role of access to knowledge, materials, and equipment as barriers and facilitators. In cases 1 and 2, participants noted initial challenges with telehealth and PPE use in GP settings due to limited prior experience. However, rapid training and individual motivation facilitated self-directed learning, enabling successful adoption. Similarly, cases 3 and 4 relied on rapid upskilling for effective redeployment to ED physiotherapy and implementation of a rapid assessment unit. These, together with technical support and learning through rapid evaluation and continuous improvement, were reported to have facilitated effective adaptation of FCPS. Telehealth tools, such as phones and eConnect software, facilitated virtual care in cases 1 and 2, while PPE availability ensured confidence in face-to-face care in cases 3 and 4, despite being perceived cumbersome as highlighted below:

Hands on stuff seems like the only way for ED physio but needs lots of PPE as well to be able to truly assess patients and sometimes, too much kit up might be difficult, attention around PPE became particularly important (Case 4 ED (Physiotherapists) R2).

Regarding implementation processes, decision-making was identified across all case sites as both a barrier and a facilitator for rapid adaptation. Initially, decisions were abrupt, top-down, and lacked clarity, leading to confusion in adapting directives for FCPS. Frequent policy changes compounded this, making implementation challenging. A GP from Case 2 noted that pre-existing poor teamwork and communication exacerbated the situation, as decisions were made without team input. Over time, leadership improved, and practitioners were granted more autonomy. A physiotherapist from Case 1 emphasised that flexible and supportive leadership empowered staff to tailor decisions to their specific FCPS needs, bolstered by knowledgeable teams and mutual support. Similarly, managers and staff in Cases 3 and 4 mentioned that later decisions used both top-down and bottom-up approaches (i.e., decisions incorporated input by senior leaders and lower-level staff), which helped respond quickly to supply needs, training, and resource allocation as noted below:

Policies later encouraged service adaptation to suit our needs (Case 3 ED (Admin staff) R7).

The two key facilitators of rapid adaptation in the outer setting revealed by our analysis were external pressure from the pandemic and financial support. The pandemic created a sense of urgency, requiring all services to adapt quickly, which led to emergency funding for healthcare. For example, a physiotherapist from case site 1 reported that government funding supported recruitment of new staff and the development and provision of new FCPS within their PC network. Similarly, administrative staff, managers and physiotherapists from cases 3 and 4 reported that the government intervened in services like the ED by providing COVID funds because surge in demand was anticipated. The fund was used to upskill staff for redeployment and the implementation of new practices as one of our participants reiterated:

As a result of COVID funding, we created a new physiotherapy service in our ED. (Case 4 ED (Clinical manager) R3)

#### Readiness for adaptation in first contact physiotherapy services delivery

Factors related to individual capability such as lack of prior training in telehealth care and contingency plans impacted the skills and confidence required for staff to provide care through virtual MSK care and function in redeployed roles, particularly in case sites 1 and 2. The uncertainty surrounding the pandemic posed an even greater challenge, as no healthcare service was fully prepared for such a large-scale event with ever-changing directives. A manager from case 3 remarked that although their ED FCPS rapidly planned in response to the pandemic, the anticipated surge in demand did not happen, resulting in possibly wasted efforts. However, pre-existing plans for new ED services in cases 3 and 4 allowed for quick implementation when resources became available while successive planning enhanced improvement.

I feel like the unknown made it really hard to plan because we had probably over-planned and didn’t end up needing it. Isn’t a bad thing because if it had gotten worse, we would have been ready. (Case 3 ED, (Manager) R2).

Similarly, in cases 1 and 2, although the early provision of equipment to facilitate remote care delivery within the GP was described as positive, the lack of a clear vision, guidance and training delayed their uptake:

Within the MSK service, we all got a laptop, we all got a mobile phone, but that wasn’t used to provide FCPS during the early phase because we weren’t sure of how to go about it. (Case 2 GP, (Physiotherapist) R1).

##### Sustainability of adaptation strategies in first contact physiotherapy services delivery

Study participants perceived adaptations rapidly implemented in FCPS as either temporary contingencies or potential permanent changes. Factors relating to the inner setting such as lack of compatibility, duplication of effort and contingencies acted as a barrier, while continuous improvement played a crucial role in facilitating the sustainability of these changes. A key challenge identified in cases 1 and 2 was the limited compatibility of telehealth with MSK care, where physical examination is essential for accurate assessment. This limitation reduced physiotherapists’ confidence in virtual consultations and may hinder their ongoing acceptability and adoption. As lockdown measures eased in 2021, public awareness around COVID-19 infection increased, and with vaccine acceptance, FCPS in cases 1 and 2 reverted mostly to in-person care. However, experience and learning demonstrated that hybrid care remained a viable option when needed, with telehealth primarily viewed as a contingency rather than a main care model:

Things have now changed back to face to face, but the pandemic taught us an extra way of working, and we still have some patients who may request a telephone consultation. (Case 1 GP (Physiotherapist) R1).

In cases 2 and 3 (a GP and ED), willing redeployed staff returned to original services, and contingency measures such as the restructuring of waiting areas were reverted. The new ED services implemented during the pandemic, which were previously desired in cases 3 and 4, remained in place though their future sustainability was uncertain due to continuous funding challenges:

COVID funding stopped a little while ago, so now we’re funding weekend cover from the physiotherapy budget, but this isn’t going to be a long-term solution. (Case 3 ED (Operational manager) R2).

## Discussion

This is the first cross-context study to examine in-depth how FCPS in primary care and emergency departments in the UK and Australia rapidly adapted during the COVID-19 pandemic, drawing on public health emergency frameworks of readiness, responsiveness, and sustainability alongside the CFIR.^19,23^ While a comparable gap in readiness for unpredictable situations such as the COVID-19 pandemic was evident across all sites, this study also identifies both shared and differing adaptation strategies implemented in response to the pandemic (Table 1), insights that may not be evident in a single-country or single-context study. Notably, telehealth use varied markedly between settings, with primary care making far greater use of telehealth than the limited uptake observed in ED during the pandemic. Overall, the findings suggest that FCPS is an adaptable model of care that demonstrates resilience in crisis conditions, offering transferable lessons for future service redesign. Specifically, this study showed that FCPS implemented the majority of rapid adaptation strategies within the first three to six months after COVID-19 was declared a global pandemic; this quick adjustment was emphasised by participants as “rapid” and an essential step to mitigate disruptions and ensure continuity in MSK care provision. However, new determinants, such as acceptability, uncertainties, duplication of effort, contingencies, opportunity for career progression and job satisfaction, not previously captured in the CFIR constructs^23^ were identified. The timeline finding adds to the literature on public health emergency response and implementation pace by demonstrating that implementation can occur more rapidly than previously documented,^4,6^ while the newly identified factors offer opportunities to broaden existing implementation frameworks.

In the absence of formal guidelines, the findings indicate that FCPS relied on available evidence and best practices, and only began to adapt once the WHO had recommended rapid adaptation.^1^ This is in line with a systematic integrative review which identified adaptation in healthcare delivery typically occurred within similar time frames during the COVID-19 pandemic.^7^ It is recognised that implementation of evidence-based interventions in health care is often prolonged and can take up to 17 years^4,6^ The COVID-19 pandemic introduced a unique dynamic, as shown in this study, with previously slow-adopted healthcare innovations such as telehealth being rapidly implemented.^8,10^ This suggests that implementation can occur much faster than has previously been reported, particularly when there is a significant shift in human attitudes and behaviours driven by an urgent need and policy. However, uncertainty around the crisis situation was identified as a factor related to the outer setting, which impacted FCPS readiness for response through rapid adaptation across contexts, as the fluidity and unpredictability of events significantly impacted planning and execution. For instance, this study identified that the rapidly changing information about the virus, treatment protocols, and public health guidelines, though helpful, also created confusion for FCPS healthcare providers and senior leaders, resulting in trial and error. This aligns with a recent systematic review highlighting how the fluid nature of crises affects planning and responsiveness.^7^ Addressing these uncertainties through clear communication and ongoing education to empower healthcare professionals and users to adapt effectively to new challenges becomes important.

Despite the unpredictable nature of the pandemic, this study identified rapid and successive planning, including continuous improvement as promising strategies across FCPS contexts. It appears that traditional implementation strategies such as prolonged planning and rigid evidence-based decision-making are less critical and less feasible in the context of a crisis, where needs are more urgent and pressing. The COVID-19 pandemic presented a unique opportunity to explore solutions to a large-scale event with available evidence.^2,3^ The critical role of continuous observation and ongoing improvement programmes in enhancing service response effectiveness during public health emergencies has previously been emphasised, particularly when evidence to guide implementation is limited at the onset of a crisis.^2,3^ Additionally, it appears that decision making during a public health emergency may depend on staff and leaders’ knowledge of risk analysis (when to act or not), and how effectively leaders communicate strategies, priorities and mobilises resources. This highlights the need for healthcare organisations to optimise health workforce capacity by establishing ongoing training on crises management, clear lines of authority, delegation of roles and responsibilities and flexible decision-making processes to respond quickly to changing circumstances.^1,7^

Implementation determinants identified outside the CFIR constructs such as acceptability, though aligned with applicable domains, offered valuable insights into additional influences on implementation during public health emergencies. This highlights a new opportunity to further refine implementation science frameworks such as CFIR. Acceptability, typically considered as an outcome of implementation, plays a crucial role in determining whether new practices are adopted by healthcare providers and patients,^5^ particularly in the context of rapid implementation. When changes are introduced quickly, stakeholder acceptance becomes a key determinant of whether new practices are adopted and sustained.^5,7^ For example, although telehealth was widely adopted in primary care and supported by government mandates for physical distancing,^7,9^ it initially met resistance from both patients and staff due to limited skills and confidence in using technology for MSK care. In contrast, the use in ED settings during the pandemic remained limited and the ongoing uptake appears slower probably due to limited evidence supporting telehealth for acute MSK presentations, making in-person care the more acceptable option, particularly in the early stages of the pandemic. Recognising these challenges and variations is vital for designing context appropriate interventions and strategies that are effective, acceptable and sustainable.

This study highlights the perceived benefits and opportunity for some adapted strategies to be sustained beyond the COVID crisis period. This was seen as essential for optimising resources post-crisis and strengthening preparedness for future emergencies. In line with WHO recommendations, some adaptations such as redeployment are likely to remain temporary, while others like telehealth have the potential to be sustained with adequate support, refinement, and evidence for long-term use.^1^ Although this study revealed that FCPS in PC largely reverted to in-person delivery post-pandemic, telehealth remained a viable option for some patients, particularly the use of hybrid models combining virtual and face-to-face care.^8^ Interestingly, evidence is beginning to emerge on the use of telehealth for MSK conditions in ED settings.^10^ Whether used as a standalone model or in hybrid form, telehealth may be challenged by the practical demands of sustaining such approaches, including additional planning, technological requirements, and the coordination needed between virtual and in-person pathways.^8,10^ This may not be a limitation of telehealth, but rather an indication that sustainable implementation requires deliberate service redesign, investment in infrastructure, clinician and patient training, and clear guidance to support appropriate use. Future research and policy should therefore focus on identifying contexts in which virtual care adds value and on developing streamlined hybrid workflows that minimise burden while maintaining accessibility and quality.

### Limitations

The study is limited to purposively selected cases from England, UK, and New South Wales, Australia. Given the similarities in FCPS functions across each country, along with commonalities in changes in other healthcare services during the COVID-19 pandemic,^7^ it is likely that the views and experiences shared by participants are not unique to these regions. However, future research is required to explore the views and experiences across different regions of each country and countries where FCPS may be less developed. Physiotherapists acted as gatekeepers, and stakeholders were identified using a snowball sampling technique, potential bias is therefore inherent to snowball technique including limited diversity of perspectives. This study set out to include patients, however, there were challenges with recruitment. While a few MSK patients initially consented to participate, they later withdrew, as a result, insights were drawn solely from practitioners, healthcare management and administrative staff. Including MSK patients would have provided a valuable perspective on how these changes were experienced during the pandemic. To minimise this limitation, participants shared their own perceptions of the impact of those service adaptations on patients, based on feedback previously shared by patients during clinical interactions. This study aligns with existing literature, though not specific to MSK patients, which has highlighted common challenges patients faced in accessing healthcare during the pandemic, including acceptability of telehealth care.^7^ Future research incorporating patient perspectives, particularly from MSK patients, could provide a more comprehensive understanding of these issues.

Triangulating interview findings with organisational policy documents detailing decisions, directives, aims, processes, and outcomes of implemented changes during the COVID-19 pandemic could have offered deeper insights into the rapid adaptation of FCPS. However, due to the burden placed on staff to locate and access these documents, coupled with the fact that participation in this activity was voluntary, participants were only able to share service plans that were readily accessible. As a result, comprehensive document analysis was not feasible, instead, the available service plans were reviewed to support our understanding of the context for the interview findings. This study is also subject to recall bias; however, the findings are in line with those reported in existing literature regarding adaptations in other healthcare services during the COVID-19 pandemic.^7^

## Conclusion

Within the first three to six months of COVID-19 being declared a global pandemic, FCPS rapidly adapted with diverse strategies across primary care and emergency care in our case study sites, with telehealth representing the most prominent context-driven variation. New factors such as acceptability, uncertainties surrounding the pandemic, duplication of effort, contingencies, opportunities for career progression, and job satisfaction emerged as influencing the speed and sustainability of the rapid adaptation of FCPS. To strengthen future responses, FCPS must be equipped for public health emergencies by developing the skills and capacity needed for rapid adaptation and contingency management, ensuring not only effective short-term responses but also sustainable long-term solutions. Policymakers could further support this by promoting and putting these findings into practice.

## Supplemental material


Supplemental material - Rapid adaptation of first contact physiotherapy services for musculoskeletal patients in the UK and Australia during the COVID-19 pandemic: A multiple case study
Supplemental material for rapid adaptation of first contact physiotherapy services for musculoskeletal patients in the UK and Australia during the COVID-19 pandemic: A multiple case study by Oluwatoyin Adenike Adeniji, Theopisti Chrysanthaki, Evangelos Pappas, Magdalena Zasada, Karen Stenner and Nicola Carey in Journal of Health Services Research & Policy.

## Data Availability

The authors confirm that the data supporting the findings of this study are available within the article and its supplementary materials.*
