# Supplementary material for: Rapid adaptation of first contact physiotherapy services for musculoskeletal patients in the UK and Australia during the COVID-19 pandemic: A multiple case study
Source: J Health Serv Res Policy. 2026 May 21;31(4):267–79. doi: 10.1177/13558196261452621 (PMC13589107; doi:10.1177/13558196261452621)
Supplement: Supplementary file 1 [file sj-pdf-1-hsr-10.1177_13558196261452621.pdf]

Journal of Health Services Research & Policy
Rapid adaptation of first contact physiotherapy services for musculoskeletal patients in the UK and Australia during the COVID-19 pandemic: A multiple case study
Adeniji OA, Chrysanthaki T, Pappas E, Zasada M, Stenner K and Carey N

Supplementary file 1: Interview guide

Interview guide for physiotherapists

Objective: Exploration of rapid adaptation in first contact physiotherapy service (FCPS) delivery in the UK and Australia during the COVID-19 pandemic.

Summary of topics

1) **About participant**
   a) How long have you been working in the practice/current hospital?
   b) For how long have you been working as a physio in the GP/emergency department?
   c) Could you tell me a bit about your role?
   d) How has your role changed during COVID-19? Why?

2) **Changes in FCPS:** How has COVID-19 affected your physiotherapy services in your GP/emergency department?
   a) What are the specific changes that occurred in your physio services (*Prompt on access, platform for delivery, caseload, patient management, structure, staff etc).*
   b) When did the "NAMED CHANGE (S)" occurred?
   c) How would you describe the way this "NAMED CHANGE (S)" was introduced in your physio services? In terms of speed (Rapid/slow) and efficiency, why?

3) **Sustainability:** Which of these change (s) have you managed to sustain? Why?

4) **Readiness:** How ready was your practice/hospital for the rapid changes that happened?

5) **Process:** Could you tell me about the process of rapid change that occurred in your physio services during the pandemic? Who was involved in making these change decisions? Why?

6) **Barriers & facilitators**
   a) Thinking all the changes you've had to make, what were the key challenges you have encountered changing your physio services?
   b) How did you address those challenges?

7) **Impact:** Can you describe how the change (s) impacted you, team member and patients?
   Patient focus Prompts:
   a) How did you manage to meet the needs/challenges of the patients visiting your physio services in your practice/hospitals during COVID-19?
   b) How responsive was your physiotherapy services during COVID-19? (Prompt based on elements)

8) **Strategies:** Thinking all the changes you've had to make, what strategies, tools and/or resources have been helpful?
   c) What worked well as your physio services changed during the pandemic?
   d) What did not work well?

Is there anything that you would like to add?
Do you have any questions for me?
Ending: Reassure confidentiality, how data will be used, gratitude for participation, further contact.

Interview guide for stakeholders

Objective: Exploration of rapid adaptation in first contact physiotherapy healthcare service (FCPS) delivery in the UK and Australia during the COVID-19 pandemic.

Summary of topics

1) **About participant**
   a) Could you tell me a bit about your role?
   b) What is your role in relation to the FCPS?
   a) Has the role developed or changed at all? When? Why?
   b) Were you here before the COVID-19 pandemic?

2) **Changes in FCPS**
   a) Could you tell me about the changes that occurred to FCPS during the pandemic?
   b) How do you think the FCPS changed during the pandemic?
   c) When did the change occurred?
   d) In terms of the rapid change and in your own opinion, what were the key challenges FCPS encountered in delivering care through "NAMED CHANGE"?
   e) What has helped FCPS address those challenges?

3) **Process of rapid change**
   a) How would you describe the process of introducing changes in a rapid way in FCPS during the pandemic?
   b) How were these decisions made?
   c) Who was involved in making these decisions and why?
   d) What was your role in changing FCPS?

4) **Views on rapid change**
   a) In general, what are your views on the rapid changes that occurred in FCPS during the pandemic?
   b) How do you feel about these changes? Why?
   c) Can you describe how the change (s) impacted you, team member and patients?
   d) In your opinion, how did FCPS manage to meet the needs/challenges of the patients visiting your practice/hospitals during COVID 19?

5) **Readiness for rapid change** *(Define readiness and present examples)*
   a) Based on your own experience, how ready was your practice/hospital in responding to the challenges posed by COVID 19 in your FCPS
   b) What other steps do you thinks are needed to be in place at your practice/hospital to ensure your FCPS are prepared to respond to rapid changes in the future?

6) **Sustainability**
   a) Which of these change (s) have FCPS managed to sustain? Why?

Is there anything that you would like to add?

Do you have any questions for me?

Ending: Reassure confidentiality, how data will be used, gratitude for participation, further contact.

Supplementary File 2: Case sites and participant's characteristics

2a: Characteristics of Selected Case Sites

Service information	Case site 1
Setting	General practice (GP) (Primary care)
Description of service provided	General practice within a primary care network where first contact physiotherapy services are integrated for the management of musculoskeletal patients.
Team structure/size	The GP has a practice manager, FCPS provided for MSK patients by a physiotherapist who works full time (37.5 hours per week) within a wider multidisciplinary team.
Service scheduling/access/referral	Traditionally, services involved a one-time appointment with an opportunity for safety netting. However, the pandemic has altered this practice, leading to patients often scheduling appointments twice, particularly when telehealth is utilised. Patient could access FCPS through GP referral, or an individual phone call to schedule appointment with the reception, and triage system. During the COVID pandemic, online self-booking became popular
Region /Country	England, United Kingdom
Contextual information	Catchment population 33,500, higher than national average on deprivation index. The practice is serving over 6,780 patients.
Study participants	n=3 (Physiotherapist, General Practitioner (GP-Doctor), and Receptionist)
	Case site 2
Setting	General practice (GP) (Primary care)
Description of service provided	General practice within a primary care networking where first contact physiotherapy services are integrated for the management of musculoskeletal patients.
Team structure/size	The GP has a practice manager and supported by physiotherapy manager. FCPS provided for MSK patients by a full-time physiotherapist who works 37.5 hours per week within a wider multidisciplinary team.
Service scheduling/access/referral	Traditionally, services involved a one-time appointment with an opportunity for safety netting. However, the pandemic has altered this practice, leading to patients often scheduling appointments twice, particularly when telehealth is utilised. Patient could access FCPS through GP referral, individual phone call to schedule appointment with the reception, triage system. During the COVID pandemic, online self-booking became popular
Country/Region	England, United Kingdom
Contextual information	Catchment population 448,787, above national average affluence index and slightly higher than average > 85-year-old population. Practice provides healthcare services for about 9200 patients.
Study participants	n=7 (Physiotherapist, General Practitioner (GP-Doctor), Practice nurse, Physiotherapy manager, Practice manager, Healthcare assistant and Receptionist)
	Case site 3
Setting	Hospital Emergency department (ED)
Description of service provided	A major teaching hospital equipped to handle complex and specialised cases, featuring a large ED where the FCPS is situated for the management of musculoskeletal patients.
Team structure/size	Three physiotherapists working in the ED, within a large and wider multi-professional team with two physiotherapists on duty each day.
Service scheduling/access/referral	The ED operates 24 hours a day, allowing patients to walk in or be brought in by ambulance. The ED employs a triage system using a scale of 1 to 5, which ranges from life threatening to minor conditions. MSK patients access the FCPS through this triage system. Additionally, physiotherapists have the option to self-select patients categorised within the triaging scale of 3 to 5, which includes those with minor injuries and MSK conditions
Country/Region	NSW, Australia
Contextual information	Catchment population of 470,000, incomes below the state average, with a slightly higher median age of 43 compared to the state average. The hospital is one of the busiest in NSW with ED presentation of around 70,000 per year.
Study participants	n=7 (Physiotherapist (n=2), Nurses (n=2), Occupational therapist, Operational manager and Admin staff)
	Case site 4
Setting	Hospital Emergency department (ED)
Description of service provided	A teaching hospital equipped to handle complex and specialised cases, with a large ED where the FCPS is situated for the management of musculoskeletal patients.
Team structure/size	Four physiotherapists work in the ED as part of a large, multi-professional team, with two physiotherapists on duty each day.
Service scheduling/access/referral	The ED operates 24 hours a day, allowing patients to walk in or be brought in by ambulance. The ED employs a triage system using a scale of 1 to 5, which ranges from life threatening to minor conditions. MSK patients access the FCPS through this triage system. Additionally, physiotherapists have the option to self-select patients categorised within the triaging scale of 3 to 5, which includes those with minor injuries and MSK conditions
Country/Region	NSW, Australia
Contextual information	Catchment population 1,3000,000, higher ethnic diversity and proportion of low incomes and young families than state average. The hospital handles 60,000 ED presentations annually,

	including 3,000 ED physiotherapy patients
Study participants	n=5 (Physiotherapist (n=2), Nurse, Clinical manager and Support staff)

Case study site no	Region	Average age of participants (years)	Respondent no	Profession	Gender	Practice duration in current role (years)	Years working in the hospital
1	England	38	1	Physiotherapist	M	4	5
			2	General Practitioner (Doctor)	M	5	5
			3	Receptionist	F	10	8
2	England	48	1	Physiotherapist	F	7	10
			2	General Practitioner (Doctor)	M	5	5
			3	Nurse	F	14	6
			4	Healthcare assistant	F	3	3
			5	Physio manager	M	5	7
			6	Practice manager	M	8	5
			7	Receptionist	F	13	3
3	NSW	44	1	Physiotherapist	F	7	10
			2	Operational manager	F	5	16
			3	Physiotherapist	M	4	7
			4	Occupational therapist	F	8	15
			5	Nurse	F	8	10
			6	Nurse	F	3	3
			7	Admin staff	F	5	11
4	NSW	42	1	Physiotherapist	M	3	3
			2	Physiotherapist	F	4	4
			3	Clinical manager	M	9	18
			4	Support staff	F	6	6
			5	Nurse	F	3	8

2b: Participant's characteristics

Supplementary file 3: Sampling Flow Chart

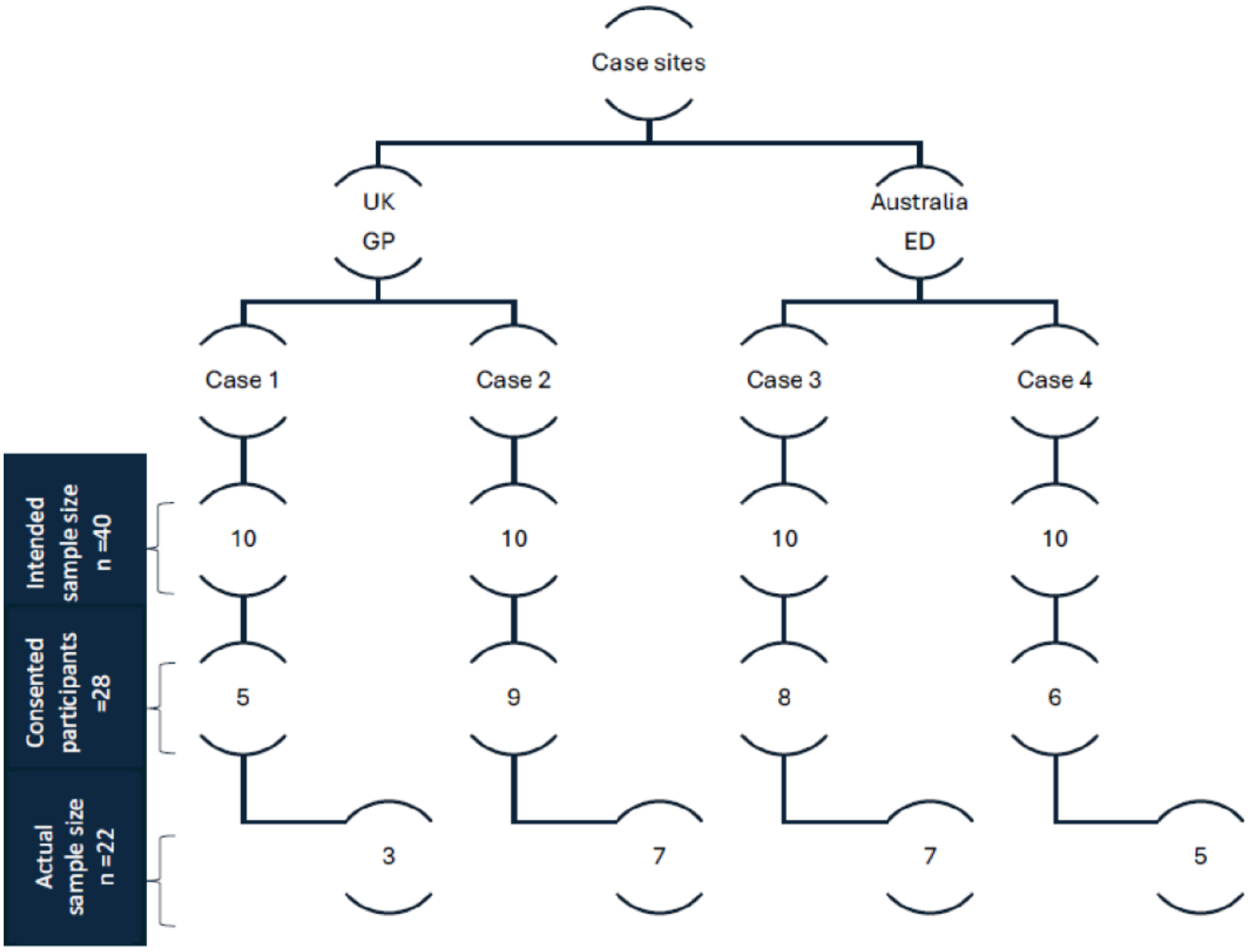

Figure 2: Case sites and participants' sampling flow chart

Supplementary file 4: Themes, Sub-themes, and Codes generated with additional quotes

Theme	Subtheme	Codes	Additional quotes not included in the text
Impacts of COVID-19 pandemic on first contact physiotherapy services	Effects on first contact physiotherapy service delivery	Disruption in service delivery	*"There were a few people left in the MSK service who were still accepting GP referrals for very urgent things. So if the GP felt there was something really, really important and urgent, then there were a couple of physios in the MSK service still doing phone appointments and the odd physical appointment as well. But that was really literally like we need somebody to help us with this. So it was very minimal and it was only the really, really kind of urgent stuff that the couple of people around us were seeing. And so, the vast majority was just, yeah, not happening at all for five to six months"* Case 2 R 1 (Physiotherapists).
		Lockdown pressure	*"Most COVID infected patients being rushed to the emergency department, people were understandably scared to come in. Many preferred to manage their musculoskeletal pain at home rather than risk contracting COVID at the ED. Plus the lockdown in effect, everyone was required to stay home, and community activities came to a halt, which likely contributed to a reduction in acute MSK pain cases. I believe these factors all played a role in the decreased presentation of MSK patients to the ED during the pandemic"* Case 3 R 3 (Physiotherapists).
	Effects on practitioners and musculoskeletal patients	Working out of scope of practice and patient complexity	*"So we were just redeployed to the community nursing team. We were basically kind of helping out with like holding a leg that a nurse was bandaging or with personal care, like cleaning people, but it wasn't what we're expert in and we didn't feel like we were making the most of our knowledge, our skills, our experience. It was really nice when we returned to our FCPS and started seeing MSK patients scan, it was like, OK, I know what I'm doing with a knee. I didn't really know what I was doing with this*

			diabetic patient who needed an insulin injection and her leg ulcers checking because that's not my area" Case 2 R1 (Physiotherapist)<br><br>*"Complexity of patients that we saw changed, rather than patients coming in with simple problems of musculoskeletal origin, or maybe one or two problems. In the area that I was working in, which is one of the most deprived area in the city and scores lowly or highly on many measures of deprivation, we were getting lots of people coming in to see FCPS because we might be the only person they've seen in six months in their practice, so they're coming in with multi joint problems, back pain, shoulder pain, hand problems. I've also got a skin rash; I've also got headaches. I've got this swollen joint so suddenly"* Case 1 R1 (Physiotherapist)
		Confusion, burn out and hazards	*"We didn't know what was happening and how to handle it, instructions changed each day, staffs were getting sick because we were exposed to COVID infection in ED"* Case 3 R1. (Physiotherapist)
First contact physiotherapy services responses to COVID-19 pandemic	Rapid adaptation for the continuity of care	Rapid adaptation strategies	*"Rapid assessment, intervention and discharge was implemented in our ED around September with a view to improve discharge planning from the ED"* Case 4 R1. (Physiotherapist)
		Rapid adaptation guidance	*"We used the advanced practice competency framework to guide upskilling staff that were redeployed to the ED, that made the process easy. And I think a guid on how to handle crises would also be helpful to facilitate adjustment process"* Case 3 R2 (Operational manager).
	Meeting musculoskeletal patient's expectations and needs	Promptness, choice, and convenience of care	*"Patients were not able to choose between in-person care and a telephone call"* Case 2 R1 (Physiotherapist)<br><br>"*Some of my patients told me telehealth was convenient when they needed only advice or not able to travel"* Case1 R1 (Physiotherapist)

		Tailored care	*"Care interventions were tailored as much as we could through telehealth, for example sending video of exercises for low back pain through email etc"* Case 1 R1 (Physiotherapist)<br><br>*"We're situated in the ED but also organise hospital services in the home"* Case 4 R 1 (Physiotherapist)
Barriers and facilitators to first contact physiotherapy services rapid adaptation	Changes in first contact physiotherapy services delivery	Barriers and facilitators relating to the inner setting, individual characteristics and outer setting and process	*"It was initially really challenging for our clinical staff delivering care virtually, and even patients receiving care because you know most people do not have the "technical know-how" and it was a general issue then, even among the GPs*" Case 2 R6 (Practice manager).<br><br>*"Definitely the lack of face to face was the biggest challenge yeah, just really hard not having someone in front of you to do an assessment. And I think the vast majority of patients wanted to actually physically see somebody. That was the theme that kept coming up as you were talking to MSK patients on the phone, seeing people on video"* Case 2 R1 (Physiotherapist)<br><br>*"There was a natural progression for me and having done it in 2020 and enjoyed it as part of the job role, I then made the move to go into it full time in 2021"* Case 1 R1 (Physiotherapist)<br><br>*"Hands on stuff seems like the only way for ED physio but needs lots of PPE as well to be able to truly assess patients and sometimes, too much kit up might be difficult, attention around PPE became particularly important"* Case 4 R2 (Physiotherapist).<br><br>*"Decisions making wasn't through our direct managers, it was a trust wide thing, and I would guess it was guided like nationally as well"* Case 1 R1 (Physiotherapist).<br><br>*"Policies kept changing, it was difficult*

			to keep up, we implemented to the best of our ability" Case 4 R3 (Clinical manager)<br><br>*"Those public health guidelines were helpful but at a point they became confusing because they were changing almost every time"* Case 1 R2 (GP-Doctor).<br><br>*"I was fortunate to work with a lovely team with varying levels of experience, some have great clinical and managerial experience while some were experienced from an IT perspective. So, everyone had something to bring to the table about how to make this work and solve problem when the management granted us autonomy in our PCN"* Case 1 R1 (Physiotherapist).<br><br>*"So, it has been something on the agenda for a long time from our Director of Allied Health for our district"* Case 3 R2 (Operational manager).
	Readiness for change in first contact physiotherapy services delivery		*"I think with hindsight, looking back, we definitely could have kept FCP services going and that would have been beneficial to those patients who needed MSK input. And but at the time, I think nobody really knew what was happening. The decisions were made with good intention and looking back now the shutdown was not helpful to us and our patients, pointing to the fact that we need to get ready for the future"* Case 1 R1 (Physiotherapist).<br><br>*"We immediately implemented our long planned rapid assessment and discharge unit for ED MSK patients as soon as we received our COVID funding"* Case 4 Respondent 3 (Clinical manager)
	Sustainability of change in first contact physiotherapy services delivery		*"We can still use telehealth for the few people that want it, maybe for initial consultation, online booking and sending exercises prescriptions through emails or applications, but we can also advise them to come in for further assessment"* Case

			1 R1 (Physiotherapist).<br>*"Well now, it's different, so when the COVID restrictions were taken away, we made the decision to reverse our proportion of telephone calls to face to face, during the pandemic we were at about 80-90% telephone call to 10ish percent face to face, we've then totally flipped it and went largely face to face"*<br>"*I think moving towards when there was vaccine, I kind of remember, that when there was some level of understanding of what the virus was, I think when vaccine came on board and we started bringing patients in, I think then yeah, then the policy changed efficiently that for examination, you could bring them in and stuff like that* Case" 2 R2 (GP-Doctor)<br><br>*"PPE wise, we started stepping down probably late last year, I'd voluntarily mask up if we feel like we need to, rather than compulsory during COVID. Space wise in ED, things have only really changed back to normal maybe this year actually. It's only this year that we've started using our short stay as short stay again for the first time in a long time. So, you know it took that long and that was only maybe a month ago or two" Case 4 R2 (Physiotherapist)*<br><br>*"You know most orthopaedic and musculoskeletal injuries that we see, we could not do through telehealth as you may need to put cast or braces on for people who survived spine fracture"* Case 3 R1 (Physiotherapist)<br><br>*"They've opened up some education pathways which used to be just for nurses, which are going to be adding up for us as well to sort of extend our skills in different areas, so that might help, have a whole lot of other range of things that we can do, like even eventually even maybe pathology, so that we can treat our patients better and even help effectively in case of crises and need*"

			Case 3 R3 (Physiotherapist)

Supplementary Material 5: Barriers and Facilitators to Rapid Adaptation in FCPS during the COVID 19 Pandemic based on CFIR Domains

Sub themes<br>CFIR domain	Facilitators	Barriers
**Adaptations in FCPS**		
Individual characteristics	*Patient acceptability of virtual care as a contingency plan	*Acceptability by staff-getting used to new experiences of virtual care (Physiotherapy needs physical examination)
	Staff willingness and motivation	*Initial and ongoing lack of acceptability of virtual care by patients
	*Opportunity for career progression	*Lack of job satisfaction
	Capability-Clinical and problem-solving skills and quick development of virtual consultation skills	Capability-Lack of telehealth skills
Inner setting	Access to knowledge and information	Lack of access to knowledge and information
	Availability of materials and equipment	Lack of appropriate equipment
	Rapid training	-
	Technical support	-
	Learning - Continuous improvement	-
	Rapid planning	
Implementation process	Staff support and teaming	Poor teamwork and communication
	*Decisions ultimately made at the local level	*Slow and uncoordinated decision making due to the changing nature of COVID directives, causing confusion
	Flexible and supportive leadership	-
	Rapid and successive planning	-
Outer setting	External pressure	-
	Financing in terms of Governments' rapid disbursement of emergency funds	-
**Readiness for Adaptation in FCPS**		
Inner setting	Availability of materials and equipment	-
		Planning and preparation
Individual characteristics	-	Lack of clinical skills and capability
Outer setting	-	*Uncertainties around the pandemic
**Sustainability of Adaptation Strategies in FCPS**		
Inner setting	-	Minimal compatibility and suitability of telehealth for MSK patients' assessment
	-	*Duplication of effort
	Learning- Organisational learning through rapid assessment and continuous improvement initiatives	-
	-	*Contingencies- Staff and patients' view of strategies such as redeployment and telehealth as contingencies
Individual characteristics	Patients and provider's acceptability	-

	Choice and convenience	-
Outer setting	Financing- Continuous availability of funding to maintain and scale up implemented adaptations	Financing- Financial challenges included the cessation of emergency funding and the absence of alternative sources to sustain new services

CFIR**:** Consolidated framework for implementation research (Damschroder et al., 2022), FCPS: First contact physiotherapy services. ***** Newly identified factors not available on CFIR constructs.
